# Cardiogenic Shock in a Young South Asian Male With Cardiomyopathy due to a Pathogenic Variant of BAG3 Gene

**DOI:** 10.1155/carm/7402283

**Published:** 2025-11-09

**Authors:** Sunil Bogati, Vasudha Maddukuri, Madhavi Kakarlapudi, Swapna Gangasani, Atul Prakash

**Affiliations:** ^1^Department of Internal Medicine, New York Medical College, St. Mary's General Hospital and St. Clare's Health, Denville, New Jersey, USA; ^2^Department of Cardiology, New York Medical College, St. Mary's General Hospital and St. Clare's Health, Denville, New Jersey, USA

## Abstract

Dilated cardiomyopathy presenting acutely as cardiogenic shock in a young adult is an infrequent and life-threatening condition. We present a case of a 25-year-old South Asian male without any significant past medical history, presenting with complaints of nonspecific abdominal bloating and subacute cough. Upon presentation, he was in sinus tachycardia and found to have moderate right pleural effusion, which was urgently drained. His echocardiogram revealed an ejection fraction of 15%–20%. He quickly progressed into cardiogenic shock with prerenal acute kidney injury, acute congestive hepatopathy, pancreatitis, and hypoxic respiratory failure. He required pressors and noninvasive positive pressure ventilation. He was transferred to a tertiary center, where he was placed on a left ventricle assist device and eventually had a heart transplant. The search for the cause of this dramatic acute dilated cardiomyopathy revealed a mutation in Bcl-2-associated athanogene 3 (BAG3).

## 1. Introduction

Cardiomyopathy refers to a group of heart diseases that impact the structure and function of the heart muscle. They are classified into dilated cardiomyopathy (DCM), hypertrophic cardiomyopathy (HCM), restrictive cardiomyopathy (RCM), and arrhythmogenic right ventricular cardiomyopathy (ARVC) [1]. DCM is a heart muscle disorder resulting from functional irregularities in cardiomyocytes. It manifests as ventricular chamber enlargement and reduces cardiac contractility. DCM can be caused by various factors, including gene mutations, viral infections, toxins (such as alcohol), mitochondrial issues, and metabolic disorders [2]. Previous estimates of DCM prevalence, based on older diagnostic methods, were 1 in 2500–3000. However, these figures likely underestimate the true prevalence. Additionally, DCM prevalence varies by gender and ethnicity [3]. Histopathological examination reveals enlarged myocytes with features of cell demise and increased myocardial fibrosis. These changes contribute to arrhythmias and heart failure. DCM is a common indication for heart transplant, yet its etiology remains unidentified in over 50% of cases. Genetic factors are increasingly recognized as significant contributors to DCM [1]. Nonischemic DCM, which is a common heart muscle pathology, is influenced by genetic factors, and the well-recognized genes that are implicated include titin (TTN) truncating variants, followed by lamin A/C (LMNA), and various desmosomal and cytoskeletal protein genes like DES, DSP, and FLNC. Accumulatively, these account for a substantial proportion of familial and sporadic cases of DCM. Additionally, some rare genes have also been identified; one of them is BCL 2-associated athanogene 3 (BAG3), which represents an important and increasingly recognized cause of DCM. These findings underscore BAG3's critical role in cardiomyocytes and the development of DCM [4].

DCM exhibits greater genetic heterogeneity compared to HCM. It involves gene mutations affecting cytoskeletal, musculoskeletal, mitochondrial, and calcium-handling proteins [1].

BAG3 genes, highly expressed in cardiac myocytes, play critical roles in maintaining sarcomere integrity, regulating macro autophagy, antiapoptosis, and mitochondrial function [4]. Its multidomain structure is made of 575 amino acids and contributes to diverse biological pathways. BAG3 mutations increase cardiac myocyte sensitivity to apoptosis under metabolic and mechanical stress [5]. Experimental studies in mice with homogenous BAG3 gene knockout revealed fulminant cardiomyopathy and skeletal myopathy due to muscle fiber degeneration with apoptosis. BAG3 mutations include missense, truncations, and frame shifts, with the E455-R477 region significantly correlated with cardiomyopathy [5]. In this case report, we present a 25-year-old male with no previous history of heart disease, initially presenting with respiratory and gastrointestinal symptoms, who was found to have severe left ventricular dysfunction, later progressing to cardiogenic shock, and the genetic testing revealed a pathogenic variant in the BAG3 gene.

## 2. Case Presentation

Our 25-year-old South Asian male graduate student, blood group B positive, with no prior medical history, then presented to the emergency department with a persistent productive cough, progressive shortness of breath on exertion, and intermittent nonradiating chest pain unrelieved by over-the-counter medications for a two-week duration. Five days before presenting, he had epigastric pain, bloating, reduced appetite, and one episode of nonbilious, nonbloody vomiting. He denied fever, chills, travel history, sick contacts exposure, nasal congestion, hemoptysis, diarrhea, or hematemesis. He used to do intermittent vaping for a year; his last use was 25 days before presentation to the hospital. He had no history of alcohol or illicit drug use, was not sexually active, and reported no family history of sudden cardiac death or cardiomyopathy.

**Figure 1:**
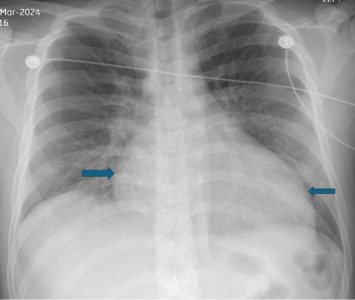
Chest X-ray image showing cardiomegaly.

**Figure 2:**
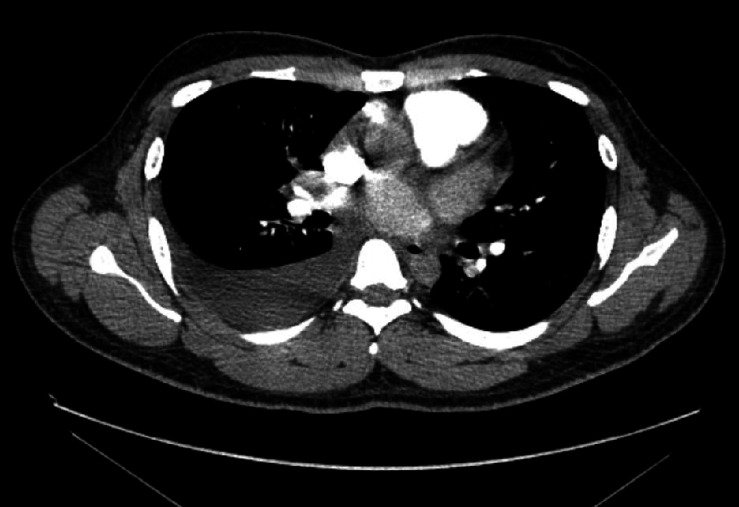
Transverse section of a CT angiogram chest showing a moderate-sized pleural effusion on the right side.

Upon examination, he was appearing ill with tachycardia, hypotension, dry mucous membranes, reduced breath sounds over the right lung base, and epigastric tenderness without jugular venous distension or peripheral edema. Initial vitals were temperature 97.3 F, HR 127/minute, respirations 20/minute, BP 96/63 mmHg, and SpO_2_ 95% on room air. Laboratory studies showed neutrophilic leukocytosis (12.3 ∗ 10^3^/mcl), mild transaminitis (AST 28 U/L, ALT 125 U/L), and a total bilirubin of 2 mg/dL. Serial high-sensitivity troponins were modestly elevated but then plateaued (34.6–38 ng/L). ECG showed sinus tachycardia with the prolonged QTc (505 ms). Chest X-ray (Figure 1) showed cardiomegaly with possible pericardial effusion; CT angiogram of chest (Figure 2) excluded pulmonary embolism but showed mild right-sided pleural effusion, patchy air space disease, gallbladder wall thickening, and mediastinal lymphadenopathy.

**Figure 3:**
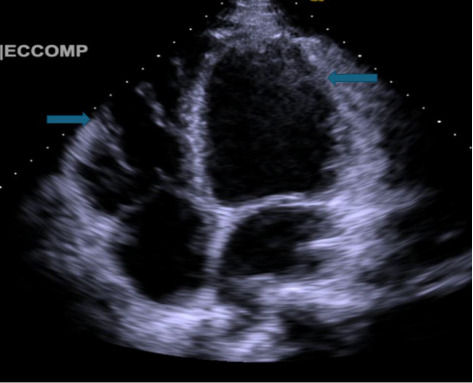
Four-chamber view on echocardiogram showing dilated left ventricle.

He was treated empirically with intravenous ceftriaxone, azithromycin, antiemetics, and intravenous fluids for presumed community-acquired pneumonia complicated by parapneumonic effusion. Thoracentesis was done, draining 600 mL of clear lymphocyte-predominant fluid. NT-proBNP was increased to 3074 pg/mL, while ESR, CRP, and procalcitonin levels were normal. Abdominal ultrasound revealed gallbladder wall thickening with trace pericholecystic fluid but no gallstones. Transthoracic echocardiography (Figure 3) revealed a severely reduced left ventricular ejection fraction of 15%–20% with global hypokinesis, severe diastolic dysfunction, mild right ventricular dilation, and a bicuspid aortic valve with leaflet calcification.

Within 12 h, the patient developed worsening epigastric pain radiating to the back with hypotension and worsening shortness of breath. Repeat CT scan of the abdomen showed acute pancreatitis, which was managed with supportive care. Despite resuscitation, he progressed to shock with metabolic acidosis, acute kidney injury, hepatic dysfunction, and hypoxemic respiratory failure, requiring vasopressors, BiPAP, and ICU transfer.

Due to continuous hemodynamic instability, he was transferred to a tertiary care center. The right heart catheterization was done, which showed right atrial pressure of 18 mmHg, right ventricular pressure of 42/10 mmHg, mean pulmonary arterial pressure of 33 mmHg, PCWP of 28 mmHg, pulmonary arterial oxygen saturation of 56%, cardiac output of 3.9 L/m, and cardiac index of 2 L/min/m^2^, consistent with severe biventricular failure. An intra-aortic balloon pump (IABP) was placed, and he also required escalating doses of inotropic agents (dobutamine, dopamine, and milrinone) along with vasopressors. Echocardiography showed persistent severe left ventricular dysfunction, with possible left ventricular apical thrombus, moderate MR, TR, and a trivial pericardial effusion. After worsening organ perfusion and low cardiac output despite maximal therapy, he was transitioned to veno-arterial extracorporeal membrane oxygenation with IABP support.

Blood cultures, autoimmune serologies, QuantiFERON-B, HIV, viral hepatitis panel, and Trypanosoma cruzi testing were all negative. Coxsackie serology was IgG positive but IgM negative, which suggested prior exposure. Genetic testing revealed a pathogenic BAG3 mutation (see supporting file 1). The heterozygous genes CRYAB and GAA were also present, but CRYAB was of uncertain significance, and the GAA variant was a benign variant. Myocarditis also could be a differential diagnosis, particularly given his preceding upper respiratory tract symptoms; however, cardiac myocardial imaging could not be performed due to instability, and myocardial histology from the left ventricular assist device or the explanted heart was not available. He ultimately underwent orthotopic heart transplantation at a regional transplant center.

## 3. Discussion

DCM has genetic (truncating TTN, LMNA, pathogenic desmosome, and BAG3 gene variants) and nongenetic (ethanol, cocaine, anthracyclines, viral and bacterial myocarditis, and peripartum cardiomyopathy) causes leading to LV systolic dysfunction, which is not explained by HTN, valvular heart disease, or CAD. These genetic mutations account for 5–15% of acquired DCM cases [6]. The incidence of DCM is more common in men than women, with onset between 20 and 60 years of age [7]. DCM is the most common indication for heart transplantation and the third most common cause of heart failure [8]. More than 50 DCM genes have been identified, which are transmitted in autosomal dominant, autosomal recessive, and matrilinear fashion, and the overlap of more than one pathogenic variant in an individual is likely to cause variable penetration and phenotypic expression [9].

Clinical presentation may vary from asymptomatic to symptomatic heart failure or sudden cardiac death [10]. Our patient presented with cardiogenic shock in the setting of a BAG3 mutation.

BAG3 gene is a 575 amino acid stress-induced protein which interacts with heat shock protein and plays an important role in autophagy and protein homeostasis. The gene is highly expressed in cardiac myocyte Z-disks and is responsible for cardiac myocyte stability and contraction [11]. Various pathogenic BAG3 variants are associated with a high risk for heart failure progression [12]. Wide phenotypic variability has been identified in carriers with DCM who eventually underwent cardiac transplantation or died from advanced heart failure [13].

Adverse outcomes with BAG3 pathogenic variants were noticed in male sex, decreased LVEF, and enlarged LVEDD, all of which were present at the time of diagnosis in our patient, who rapidly deteriorated and developed cardiogenic shock needing inotropes [14, 15].

BAG3 mutations, namely, Arg218Trp, causing DCM, are in exon 3, and heart transplantation was needed in a patient at 20 years of age who had the Pro209Leu mutation [16]. There is also data of three severely affected males with the DCM phenotype in a four-generation pedigree who underwent cardiac transplant, and their whole exome sequencing showed a deletion in exon 4 of BAG3 [17]. Our patient was also found to have a BAG3 mutation and had a heart transplant at 25 years of age for DCM.

Human genomic studies also confirmed that DCM-associated BAG3 mutations result in truncation and complete loss of BAG3 function with an incidence of 6.7% of all DCM cases [18]. This is further supported by BAG3 knock-out mice and zebrafish models developing DCM shortly after birth [19].

Clinically, BAG3 mutations have diverse phenotypic presentations with a mean age of diagnosis at 36.9 years, with early onset in men (at around 30 years); however, our patient presented even earlier at 25 years of age. An echocardiogram is vital, and having a low LVEF along with NYHA class III-IV is associated with a higher incidence of death or need for heart transplant. It was also observed that 30.1% of patients needed an LV assist device and heart transplant [20]. Our patient also needed an emergent LV assist device with an eventual cardiac transplant. In addition, myocarditis and acute onset DCM in BAG3 carriers may be precipitated by viral infections through P38 signaling pathways, causing inflammation and apoptosis [4]. Our patient had upper respiratory tract symptoms, which occurred before fulminant heart failure, which raises the possibility that viral infection triggered the genetic vulnerability. It is important to understand that this rapid decline was not only due to genetic mutation alone. Acute severe pancreatitis during hospitalization could also have provoked a systemic inflammatory response via a massive cytokine cascade. This inflammatory burden can cause distributive shock and myocardial depression, adding to the already limited myocardial reserve. The absence of overt signs of cardiogenic shock, like jugular venous distension and peripheral edema, further suggests the possibility of a mixed picture, in which systemic inflammation or early sepsis contributed to the circulatory collapse.

Overall, our patient's presentation is multifactorial. The already vulnerable myocardium from BAG3-associated DCM, aggravated by a potential viral trigger and acute systemic inflammation from pancreatitis, could have caused this presentation. The adverse outcomes in the setting of BAG3 mutation highlight the significance of DNA sequencing to identify high-risk individuals. This case underlines the need to think of overlapping mechanisms.

Also, LV reverse remodeling has a strong correlation with prognosis in DCM patients, which usually takes 6 months to 1 year of appropriate goal-directed treatment. In the present era of precision and genomic medicine, adverse events related to BAG3 mutations may be prevented by identifying high-risk individuals by DNA sequencing and early intervention with guideline-directed medical therapy.

## 4. Conclusion

DCM presenting as cardiogenic shock when identified in any young individual should prompt clinicians to think about genetic etiology, in particular BAG3 genetic mutations, which are being increasingly identified in the recent genomic era. Such patients may need urgent or emergent heart transplants and expert cardiac care.

## Data Availability

All relevant clinical details are included within this manuscript. Additional information can be obtained from the corresponding author upon reasonable request, while maintaining patient confidentiality.

## Supporting Information

Supporting file 1-BAG3 mutation-genetic testing.
