# Supplementary material for: Cardiogenic Shock in a Young South Asian Male With Cardiomyopathy due to a Pathogenic Variant of BAG3 Gene
Source: Case Rep Med. 2025 Nov 9;2025:7402283. doi: 10.1155/carm/7402283 (PMC12620047; doi:10.1155/carm/7402283)
Supplement: Supporting Information — Additional supporting information can be found online in the Supporting Information section. [file 7402283.f1.pdf]

Reason for testing

Not provided

Test performed

Sequence analysis and deletion/duplication testing of the 82 genes listed in the Genes Analyzed section.

- Invitae Cardiomyopathy Comprehensive Panel

RESULT: POSITIVE

One Likely Pathogenic variant identified in BAG3. BAG3 is associated with a spectrum of autosomal dominant cardiac and neuromuscular conditions.

Additional Variant(s) of Uncertain Significance identified.

GENE	VARIANT	ZYGOSITY	VARIANT CLASSIFICATION
BAG3	c.1417C>T (p.Arg473*)	heterozygous	Likely Pathogenic
CRYAB	c.319C>T (p.Arg107Cys)	heterozygous	Uncertain Significance
GAA	c.2065G>A (p.Glu689Lys)	heterozygous	Benign (Pseudodeficiency allele)

About this test

This diagnostic test evaluates 82 gene(s) for variants (genetic changes) that are associated with genetic disorders. Diagnostic genetic testing, when combined with family history and other medical results, may provide information to clarify individual risk, support a clinical diagnosis, and assist with the development of a personalized treatment and management strategy.

Clinical comments

- When a single Variant of Uncertain Significance is found in a requisitioned gene that is only associated with autosomal recessive condition(s), it may not be included in the report.

shogati@ph
69.112.12.

Clinical summary

A likely pathogenic variant, c. 1417C>T (p.Arg473*), was identified in BAG3.

- The BAG3 gene is associated with autosomal dominant dilated cardiomyopathy (DCM) (MedGen LID: 462643), myofibrillar myopathy 6 (MFM6)
- This result is consistent with a predisposition to, or diagnosis of. BAG-related conditions
- DCM is defined by left ventricular dilation and systolic dysfunction. Symptoms include palpitations, dizziness, syncope, chest pain, shortness of breath, heart failure and, in some cases, sudden cardiac arrest death (PMID: 75068017. 755840161. For more information about diganosis and management of DCM, please visit Invitae's website at www.invitae.com/management-guidelines. MFM6 is an early-onset, rapidly progressive form of myofibrillar myopathy (PMID: 22714908). Unlike other myofibrillar myopathies, MFM6 presents in childhood and is characterised by rapid progression of limb and axial muscle weakness, cardiomyopathy and respiratory insufficiency (PMID: 19035932, 23622353). Significant axonal neuropathy with the presence of giant axons on nerve biopsy has also been reported (PMID: 22734908). BAG3-related Charcot-Marie-Tooth disease presents with adult-onset sensorimotor axonal neuropathy in the absence of myopathy (PMID: 287546661).
- Biological relatives have a chance of being at risk for BAG3-related conditions and should consider testing if clinically appropriate.

A Variant of uncertain significance, c.319C>T (p.Arg107Cys), was identified in CRYAB.

- The CRYAB gene is associated with autosomal dominant and recessive cataracts (MedGen UID: $14707). It is also associated with autosomal
  dominant and recessive myofibrillar myopathy 2 (MFM2) (Medoen UID: 324255). Additionally, the CRiAB gene has preliminary evidence supporting a correlation with autosomal dominant dilated cardiomyopathy (DCM) (MedGen UID: 767563).
- Not all variants present in a gene cause disease. The clinical significance of the variant(s) identified in this gene is uncertain. Until this uncertainty can be resolved, caution should be exercised before using this result to inform clinical management decisions.
- Complimentary familial VUS testing is not offered. Details on our VUS Resolution and Family Variant Testing Programs can be found at
  https://www.irwitae.com/farrilly

Variant details

BAG3, Exon 4, c. 1417C>T (p.Arg473*), heterozygous, Likely Pathogenic

- This sequence change creates a premature translational stop signal (p.Arg473*) in the BAG3 gene. While this is not anticipated to result in nonsense-mediated decay, it is expected to disrupt the last 103 amino acid(s) of the BAG3 protein.
- This variant is not present in population databases (gnomAD no frequency).
- This premature translational stop signal has been observed in individuals with dilated cardiomegaly (PMID: 28436997. 32160020).
- ClinVar contains an entry for this variant (Variation ID: 620529).
- This variant disrupts a region of the BAG3 protein in which other variant(s) (p.Arg477His) have been observed in individuals with BAG-related conditions (PMID: 21353195). This suggests that this is a clinically significant region of the protein and that variants that disrupt it are likely to be disease-causing.
- In summary, the currently available evidence indicates that the variant is pathogenic, but additional data are needed to prove that conclusively.
  Therefore, this variant has been classified as likely pathogenic.

CRYAB, Exon 3, c. 319C>T (p.Arg107Cys), heterozygous, Uncertain Significance

- This sequence change replaces arginine, which is basic and polar, with cysteine, which is neutral and slightly polar, at codon 107 of the CRYAB protein (p.Arg107Cys).
- This variant is present in population databases (rs782520163, BRomAD 0.007%).
- This variant has not been reported in the literature in individuals affected with CRYAB-related conditions.
- ClinVar contains an entry for this variant (Variation D: 1410499).
- An algorithm developed to predict the effect of missense changes on protein structure and function (PolyPhen-2) suggests that this variant is
- In summary, the available evidence is currently insufficient to determine the role of this variant in disease. Therefore, it has been classified as a Variant of uncertain significance.

GAA, Exon 15, c.2065G>A (p.Clu689Lys), heterozygous, Benign (Pseudodeficiency allele)

- This sequence change replaces glutamic acid, which is acidic and polar, with lysine, which is basic and polar, at codon 689 of the GAA protein (p.Glu689Lys)
- This variant is present in population databases (rs1800309, gnomAD 2496), including at least one homozygous and/or hemizygous individual.
- The GAA gene is associated with autosomal recessive glycogen storage disease type II (GSDII, also known as Pompe disease (MedGen UID 5340). This variant is a known pseudodeficiency allele (PMID: 18301443, 21644219, 1780574, 20080426), and individuals with this variant can exhibit acid alpha-glucosidase deficiency during enzyme analysis. Individuals with pseudodeficiency alleles may also exhibit positive results on related biochemical tests, but pseudodeficiency alleles are not known to cause disease. Although

pseudodeficiency alleles do not cause disease; other carrier relatives may have abnormal enzyme testing. In addition, it has been observed in many individuals who did not have clinical evidence of Pompe disease (PMID: 20080426, 18301443) -

- ClinVar contains an entry for this variant (Variation ID: 4030)
- Advanced modelling of protein sequence and biophysical properties (such as structural, functional, and spatial information, amino acid conservation, physicochemical variation, residue mobility, and thermodynamic stability) performed at Invitae indicates that this missense variant is not expected to disrupt GAA protein function with a negative predictive value of 95%6.
- For these reasons, this variant has been classified as a benign pseudodeficiency allele.

Genes analyzed

This table represents a complete list of genes analyzed for this individual, including the relevant gene transcript(s). If more than one transcript is listed for a single gene, variants were reported using the first transcript listed unless otherwise indicated in the report. An asterisk (*) indicates that this gene has a limitation. Please see the Limitations section for details. Results are negative unless otherwise indicated in the report. Benign and likely benign variants are not included in this report, and in specific scenarios, variants of uncertain significance in the requisitioned panel may not be included in this report.

GENE	TRANSCRIPT
ABCC9	NM_005691.3
ACADVL	NM_000018.3
ACTC1	NM_005159.4
ACTN2	NM_001103.3
AGL	NM_000642.2
ALMS1	NM_015120.4
ALPK3	NM_020778.4
BAG3	NM_004281.3
BRAF	NM_004333.4
CACNA1C	NM_000719.6;NM_00112984 0.1
CBL	NM_005188.3
CPT2	NM_000098.2
CRYAB	NM_001885.2
CSRP3	NM_003476.4
DES	NM_001927.3
DMD	NM_004006.2
DNAJC19	NM_145261.3
DOLK	NM_014908.3
DSC2	NM_024422.4
DSG2	NM_001943.3
DSP	NM_004415.2
ELAC2	NM_018127.6
EMD	NM_000117.2
EYA4	NM_004100.4
FHL1	NM_001449.4
FKRP	NM_024301.4
FKTN	NM_001079802.1
FLNC*	NM_001458.4
GAA	NM_000152.3
GLA	NM 000169.2
HCN4	NM_005477.2
HRAS	NM_005343.2
JUP	NM_002230.2
KRAS	NM_004985.4
LAMP2	NM_002294.2

GENE	TRANSCRIPT
LMNA	NM_170707.3
LZTR1	NM_006767.3
MAP2K1	NM_002755.3
MAP2K2	NM_030662.3
MRAS	NM_012219.4
MTO1	NM_012123.3
MYBPC3	NM_000256.3
MYH7	NM_000257.3
MYL2	NM_000432.3
MYL3	NM_000258.2
MYLK3	NM_182493.2
NF1*	NM_000267.3
NRAS	NM_002524.4
PCCA	NM_000282.3
PCCB	NM_000532.4
PKP2	NM_004572.3
PLN	NM_002667.3
PPCS	NM_024664.3
PPP1CB	NM_206876.1
PRKAG2	NM_016203.3
PTPN11	NM_002834.3
RAF1	NM_002880.3
RASA1	NM_002890.2
RBM20	NM_001134363.2
RIT1	NM_006912.5
RYR2	NM_001035.2
SCN5A	NM_198056.2
SDHA*	NM_004168.3
SGCD	NM_000337.5
SHOC2	NM_007373.3
SLC22A5	NM_003060.3
SOS1	NM_005633.3
SOS2	NM_006939.2
SPRED1	NM_152594.2
TAZ	NM_000116.4

GENE	TRANSCRIPT
TBX20	NM_001077653.2
TCAP	NM_003673.3
TMEM43	NM_024334.2
TMEM70	NM_017866.5
TNNC1	NM_003280.2
TNNI3	NM_000363.4
TNNI3K	NM_015978.2
TNNT2	NM_001001430.2
TPM1	NM_001018005.1
TTN*	NM_001267550.2
TTR	NM_000371.3
VCL	NM_014000.2

METHODS

- Genomic DNA obtained from the submitted sample is enriched for targeted regions using a hybridization-based protocol, and sequenced using Illumina technology Unless otherwise indicated, all targeted regions are sequenced with a 50x depth or are supplemented with additional analysis.
  Reads are aligned to a reference sequence (GRCh37), and sequence changes are identified and interpreted in the context of a single clinically relevant transcript, indicated in the Genes Analyzed table. Enrichment and analysis focus on the coding sequence of the indicated transcripts. 20bp of flanking intronic sequence, and other specific genomic regions demonstrated to be causative of disease at the time of assay design. Promoters, untranslated regions, and other non-coding regions are not otherwise interrogated. For some genes only targeted loci are analyzed Exonic deletions and duplications are called using an in-house algorithm that determines copy number at each target by comparing the read depth for each target in the proband sequence with both mean read depth and read depth distribution, obtained from a set of clinical samples Markers across the X and Y chromosomes are analyzed for quality control purposes and may detect deviations from the expected sex chromosome complement. Such deviations may be included in the report in accordance with internal guidelines. Variants are reported according to the Human Genome Variation Society (HCVS) guidelines. Confirmation of the presence and location of reportable variants is performed as needed based on stringent criteria using one of several validated orthogonal approaches (PubMed ID 30610921). Sequencing is performed by Invitae Corporation (1400 16th Street, San Francisco, CA 94103, #05D2040778). Confirmatory sequencing is performed by Invitae Corporation (1400 16th Street, San Francisco, CA 94103, #05D2040778). RNA sequencing is performed by Invitae Corporation (1400 16th Street, San Francisco, CA 94103, #0SD2040778)

  The following additional analyses are performed if relevant to the requisition. For PMS2 exons 12-15, the reference genome has been modified to force all sequence reads derived from PMS2 and the PMS2CL pseudogene to align to PMS2, and variant calling algorithms are modified to support an expectation of 4 alleles. If a rare SNP or indel variant is identified by this method, both PMS2 and the PMS2CL pseudogene are amplified by long-range PCR and the location of the variant is determined by Pacific Biosciences (PacBio) SMRT sequencing of the relevant exon in both long-range amplicons. If a CNV is identified, MLPA or MLPA-seq is run to confirm the variant. If confirmed, both PMS2 and PMS2CL are amplified by long-range PCR, and the identity of the fixed differences between PMS2 and PMS2CL are sequenced by PacBio from the long-range amplicon to disambiguate the location of the CNV. For C9orf72 repeat expansion testing, hexanucleotide repeat units are detected by repeat-primed PCR (RP-PCR) with fluorescently labeled primers followed by capillary electrophoresis. Interpretation Reference Ranges: Benign (Normal Range): <25 repeat units, Uncertain: 25-30 repeat units, Pathogenic (Full Mutation): >=31 repeat units (MID: 21944779, 22406228, 23111906, 28689190, 31315673, 33168078, 33575483). A second round of RP-PCR utilizing a non-overlapping set of primers is used to confirm the initial call in the case of suspected allele sizes of 22 or more repeats. For RNA analysis of the genes indicated in the Genes Analyzed table, complementary DNA is synthesized by reverse transcription from RNA derived from a blood specimen and enriched for specific gene sequences using capture hybridization, After high-throughput sequencing using Illumina technology, the output reads are aligned to a reference sequence (genome build GRCh37; custom derivative of the RefSeq transcriptore) to identify the locations of exon junctions through the detection of split reads. The relative usage of exon junctions in a test specimen is assessed quantitatively and compared to the usage seen in control specimens. Abnormal exon junction usage is evaluated as evidence in the Sherloc variant interpretation framework. If an abnormal splicing pattern is predicted based on a DNA variant outside the typical reportable range, as described above, the presence of the variant is confirmed by targeted DNA sequencing.
- A PMID is a unique identifier referring to a published, scientific paper. Search by PMID at http://www.ncbi.nlm.nih.gov/pubmed.
- An rsID is a unique identifier referring to a single genomic position and is used to associate population frequency information with sequence changes at that position. Reported population frequencies are derived from a number of public sites that aggregate data from large-scale population sequencing projects, including ExAC (http://exac.broadinstitute.org). gnomAD (http://gnomad.broadinstitute.org), and dbSNP (http://ncbi.nlm.nih.gov/SNP)

- A MedGen ID is a unique identifier referring to an article in MedGen, NCBI's centralised database of information about genetic disorders and phenotypes. Search by MedGen ID at http://www.ncbi.nlm.nih.gov/medgen. An OMIM number is a unique identifier referring to a comprehensive entry in Online Mendelian Inheritance in Man (OMIM). Search by OMIM number at http://omim.org/
- Invitae uses information from individuals undergoing testing to inform variant interpretation. If "Invitae" is cited as a reference in the variant details, this may refer to the individual in this requisition and/or historical internal observations.

LIMITATIONS

Based on validation study results, this assay achieves >99% analytical sensitivity and specificity for single nucleotide variants, insertions and deletions <15bp in length, and exon-level deletions and duplications. Invitae's methods also detect insertions and deletions larger than 15bp but smaller than a full exon but sensitivity for these may be marginally reduced. Invitae's deletion/duplication analysis determines copy number at a single exon resolution at virtually all targeted exons. However, in rare situations, single-exon copy number events may not be analyzed due to inherent sequence properties or isolated reduction in data quality. Certain types of variants, such as structural rearrangements (eg inversions, gene conversion events, translocations, etc.) or variants embedded in sequences with complex architecture (eg. short tandem repeats or segmental duplications), may not be detected.

Additionally, it may not be possible to fully resolve certain details about variants, such as mosaicism, phasing, or mapping ambiguity. Unless explicitly guaranteed, sequence changes in the promoter, non-coding exons, and other non-coding regions are not covered by this assay. Please consult the test definition on our website for details regarding regions or types of variants that are covered or excluded for this test. This report reflects the analysis of an extracted genomic DNA sample. While this test is intended to reflect the analysis of extracted genomic DNA from a referred patient, in very rare cases the analyzed DNA may not represent that individual's constitutional genome, such as in the case of a circulating hematolymphoid neoplasm, bone marrow transplant, blood transfusion, chimerism, culture artifact or maternal cell contamination. Interpretations are made on the assumption that any clinical information provided, including specimen identity, is accurate. Invitae's RNA analysis is not designed for use as a stand-alone diagnostic method and cannot determine absolute RNA levels. Results from the RNA analysis may not be informative for interpreting copy number events. Additionally, sensitivity to detect RNA splicing events may be reduced for variants in the first donor site of each gene.

NF1: Sequencing analysis for exons 2, 7, 25, 41, 48 includes only cds +/- 10 bp. SDHA: Deletion/duplication analysis is not offered for this gene and sequencing analysis is not offered for exon 14. Sequencing analysis for exons 6-8 includes only cds +/- 10 bp. TTN: Exons 45-46, 147, 149, 164, 172-201 (NM_001267550.2) are excluded from analysis. TTN variants are included in the primary report based on functional effect and/or location. A complete list of variants of uncertain significance, likely benign and benign variants in TTN is available upon request. Variants are named relative to the NM_001267550.2 (meta) transcript. Variants in the coding sequence and intronic boundaries of the clinically relevant NM_133378.4 (NA) and fetal isoforms are reported (PMID: 25589632, 29598826, 29691892, 31660661), with the exception of the PEVK tandem repeat region (172-198) (PMID: 28040389). FLNC: Deletion/duplication analysis is not offered for exon 47. Sensitivity and specificity for single nucleotide variants, insertions and deletions in exons 47-48 may be reduced due to the presence of segmental duplications overlapping the region.
